# The Use of Polymer Membranes to Counteract the Risk of Environmental of Soil and Water Contamination

**DOI:** 10.3390/membranes11060426

**Published:** 2021-06-04

**Authors:** Anna Rabajczyk, Maria Zielecka, Krzysztof Cygańczuk, Łukasz Pastuszka, Leszek Jurecki

**Affiliations:** Scientific and Research Center for Fire Protection—National Research Institute, Nadwiślańska 213, 05-420 Józefów, Poland; mzielecka@cnbop.pl (M.Z.); kcyganczuk@cnbop.pl (K.C.); lpastuszka@cnbop.pl (Ł.P.); ljurecki@cnbop.pl (L.J.)

**Keywords:** polymer membranes, application, CBRN contamination, remediation

## Abstract

Chemical, biological, radiological, or nuclear (CBRN) contamination of the environment is a significant threat to human health and life as well as environmental safety. It is then necessary to take actions aimed at minimizing and eliminating the threat. Depending on the type of contamination, various methods are used, including sorption, biodegradation, separation, or ion exchange processes in which membranes play an important role. The type of membrane is selected in respect of both the environment and the type of neutralized pollutants. Therefore, the production and modification of membranes are being adapted to the type of contamination and the purpose of the work. This article presents examples of membranes and their possible applications depending on the part of the environment subject to reclamation and the type of contamination.

## 1. Introduction

The environment is a very dynamic system which is shaped by various factors, including temperature, pressure, water or wind erosion processes, pH, redox potential, and human activity. Various substances introduced by humans to the environment may determine changes taking place in nature, affecting organisms, disrupting their natural functions. Contamination occurs when a dangerous substance is introduced into the environment or present in a given element of the environment in concentration, form, or nature, that violates the natural system of the environment, exceeds the applicable regulations, and poses a threat to human health. It should be added that contamination of the environment may be caused by natural processes taking place in the environment, such as volcanic eruptions, but most often it is caused by human activity, such as technological line failure, sewage discharge, leachate from landfills, and incineration in uncontrolled conditions.

Depending on the source of emission and the type of contamination, the migration routes in the environment and the impacts on individual elements may be different. The variety and number of sources of pollutant emissions to the environment and the type of the introduced substance can often cause irreversible or almost irreversible changes in nature. Therefore, chemical, biological, radiological, or nuclear (CBRN) events require appropriate measures to minimize their negative impact.

**Figure 1:**
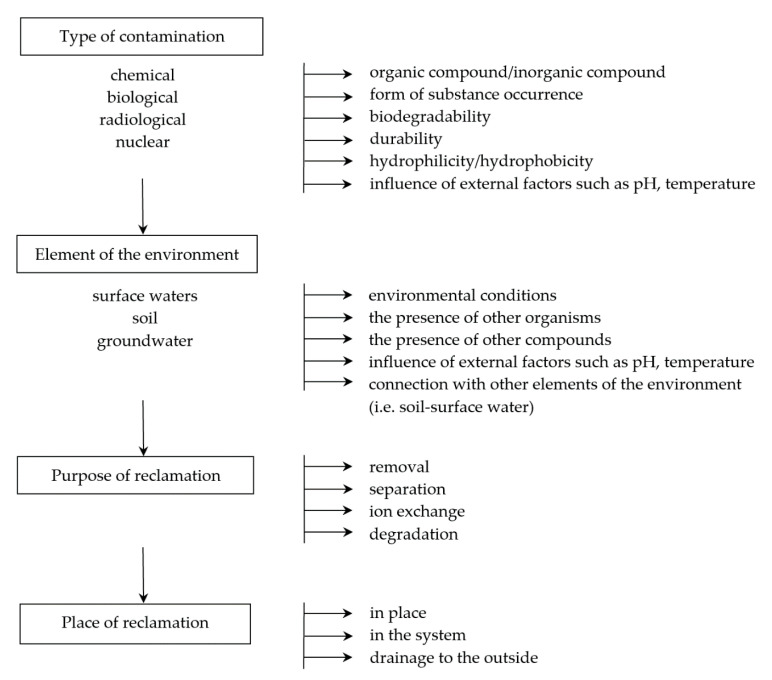
An example of a decision-making path necessary for the correct selection of the technique and type of membrane.

In the case of contamination, it is very important to select the appropriate techniques and tools that minimize the negative impact and make it possible to remove the substance or organism (in the case of biological contamination) from the system. Products that may be generated during environmental remediation should also be considered so that they do not present a greater risk than the substance to be neutralized. Tools and techniques must therefore be adapted to the type of environment, taking into account physicochemical and biological conditions, including diffusion process, temperature and pressure conditions, as well as the natural presence of organisms (Figure 1).

The aim of the article is to present the potential applications of polymer membranes in situations of environmental contamination, including soil, surface water, and groundwater. The presented data will allow for the appropriate selection of membranes depending on the type of environment contaminated and the type of removed substances. Properly carried out activities in this area determine not only the safety of the environment, but also the safety of human life and health. The literature review was undertaken using the following databases: Web of Knowledge, Scopus, Google Scholar.

## 2. Application of Membranes in Contamination Situations

Potential emerging pollutants, such as hazardous chemicals, toxic metals, bio-waste, etc., pose a serious threat to health, hygiene, and ecology by polluting the environment. These pollutants from various sources, including industrial wastewater, mainly from the pharmaceutical, food, and metal processing industries, can contaminate water and disrupt aquatic ecosystems. The discharged wastewater at the source requires clear identification, separation and disposal, otherwise, it can pose serious problems for water quality and ecology in general. Conventional water treatment methods, such as adsorption, bio-oxidation, coagulation, sedimentation, and filtration as well as hybrid methods, such as chlorination and UV irradiation, have been widely described in the literature, although most of these approaches are insufficient for effective wastewater treatment [1].

**Figure 2:**
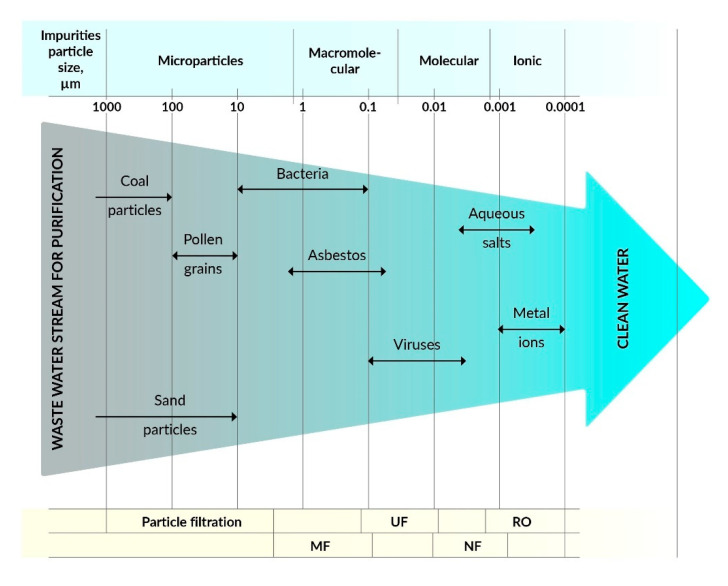
Dependence of filtration methods on the type of pollutants.

On the other hand, water treatment by membrane-based separation processes is quite expensive and energy-intensive compared to other conventional treatment technologies. However, membrane treatment processes have several clear advantages, such as the production of high-quality water with a high recovery rate of valuable chemicals/metals and low maintenance costs [2,3]. The most commonly used methods are microfiltration (MF), ultrafiltration (UF), nanofiltration (NF), reverse osmosis (RO), and forward osmosis (FO), and some hybrid technologies such as membrane bioreactors (MBR) and photocatalytic membrane reactors (PMR). Membrane processes depend on the type of membranes that are made of various materials, including polymers, ceramics, zeolites, etc., with specific filtration properties. The effectiveness of membranes depends on the surface charge, pore size, membrane morphology, and hydrophobic/hydrophilic properties. Polymer membranes can be used in various filtration methods, such as MF, UF, NF, RO, and FO depending on pore size and morphology as well as specific separation needs (see Figure 2).

The above filtration methods use various separation techniques, e.g., solution diffusion or molecular diffusion or the size exclusive principle [4].

The membranes used for MF have larger pore sizes (0.1–5 µm) than the membranes used for UF, which are typically used to separate contamination with a particle size in the range of 0.1–10 µm. On the other hand, UF membranes with pore sizes from 0.01 to 0.1 μm can be used to separate colloidal particles, macromolecules, biopolymers, and viruses, whose sizes are usually in the range from 0.01 to 0.2 μm, and the process uses the principle of size exclusion. Commercially, UF is widely used for wastewater treatment, recovery of surfactants from industrial line washing, and in food processing and protein separation, etc. UF membranes are made of cellulose derivatives, inorganic materials, such as TiO_2_, Al_2_O_3_, ZrO, as well as from common polymers, such as poly(acrylonitrile) (PAN), poly(sulfonamide) (PSA), poly(ether sulfone) (PES), poly(vinylidene fluoride) (PVDF), etc. [5,6].

NF membranes enable the separation of particles in the size range of 0.001–0.01 µm, which include most organic compounds, biomacromolecules, and various metal salts (except divalent salts). The capacity of NF is between RO and UF [7]. RO membranes are non-porous, made of solid polymers with voids, free-volume channels, or pore sizes ranging from ∼0.0001 to 0.001 μm [8]. RO membranes separate low molecular weight inorganic components, including metal ions. The most common applications of RO are the treatment of wastewater from pulp and paper mills to produce drinking water [9].

In recent years, ceramic or zeolite composite membranes have emerged as high-performance RO and NF membranes that have been successfully commercialized for the separation of pollutants [10]. These membranes are generally made of composite materials and contain various fibers such as hollow or structural fibers, or sheet nanostructures such as graphene and layered silicates in the polymer matrix [11]. Several problems with the use of RO membranes, such as their high energy requirements and fouling of membrane surfaces, have resulted in the development of techniques [12] and FO membranes in which the osmotic gradient across the membrane plays an important role in mass transport and separation [13]. FO is more suitable and energy efficient for the treatment of membrane fouling wastewater (e.g., landfill leachate) which may not be economical for RO. Initially, FO was regarded as an effective pretreatment step for subsequent processes in which purified water could be recovered from the dilute solution [14]. However, FO as a single filter technique also finds some niche applications, such as diluting fertilizers and thickening fruit juices [15].

### 2.1. Surface Water Contamination

Water pollution is a widespread problem worldwide, and the sources of pollution can be geological or anthropogenic [16]. The types and concentrations of natural pollutants depend on the type of geological materials through which groundwater flows and the type of catchment development. Surface waters moving through different terrains can contain various substances such as magnesium, calcium, chloride, arsenate, fluorine, nitrate, and iron [17,18]. If naturally occurring elements are present at unacceptable levels, water may be contaminated [19]. Other contaminants are by-products made by humans, industries, and agriculture, including heavy metals, mercury, copper, chromium, lead, and hazardous chemicals, dyes, and compounds such as insecticides and fertilizers. Generally, there are four types of surface water pollution, i.e., inorganic, organic, biological, and radiological.

Significant natural contamination of surface water is affected by calcium and magnesium compounds causing water hardness. Other pollutants coming from natural sources, industrial processes, as well as from water supply systems are compounds of fluorine, arsenic, lead, copper, chromium, mercury, antimony and cyanides [20]. The main anthropogenic sources of organic pollutants are pesticides, household waste, industrial waste, etc. [21]. Contamination with organic materials can cause serious health problems, such as cancer, endocrine disruptions, and nervous system disruption [22]. Biological water contamination is caused by the presence of living organisms such as algae, bacteria, protozoa or viruses [23]. Each of them can cause different problems in the water.

Radiological contamination is caused by radioactive elements. Sources of radioactive material can be soil or rocks through which water flows or some industrial waste [24]. The erosion of natural deposits of certain (radioactive) minerals can emit radiation (like α and β). At the same time, radioactive elements, such as U^226^, Ra^226^, Ra^228^, and Rn^228^ seem to be a bigger problem in groundwater than in surface water. All types of radiological contamination increase the risk of cancer [25].

Depending on the type of contaminants present in the water, they are removed by various methods using both physical and chemical processes. Some of the common methods of water purification are precipitation and coagulation (including water softening and removal of heavy metals, phosphorus, fluoride, arsenic, dyes), distillation, adsorption (often using activated carbon, zeolites, silica gel, or ion exchange resins). Often, depending on the type of contaminants present in the water, hybrid treatment processes combining different methods are also used. Such activities are aimed at obtaining waters of appropriate purity, taking into account not only the efficiency of operation, but also the costs of these processes.

Currently, innovative membrane technologies are used more and more often for water purification. In water treatment, the best results are achieved by pressure processes, e.g., reverse osmosis, or electrically driven processes, e.g., electrodialysis (ED) [26]. The basic principle of membrane separation using electrodialysis is similar to the ion exchange reaction [27].

Polymer membranes used in osmotic processes generally have an open porous support layer and a thin, less porous skin layer of the same material. Separation takes place in the epidermis layer, and the carrier is easily permeable to water and substances undissolved in water. In MF/UF membranes, the active part of the membrane is a selective surface layer with pores from 0.01 to 0.2 mm, which is responsible for the efficiency of the filtration process. MF and UF membranes are most often made of polymers, e.g., polysulfone (PSF), polyethersulfone (PES), polyacrylonitrile (PAN), polypropylene (PP), polytetrafluoroethylene (PTFE), and polyvinylidene fluoride (PVDF). These polymers exhibit excellent permeability, selectivity, and stability (chemical, mechanical, and thermal) and are therefore used in water treatment. PSF and PES are mainly used as membrane materials used in UF, NF and RO [28]. In recent years, research on the improvement of membranes has focused on introducing nanomaterials into the structure of the membrane. Advanced nanocomposite membranes can be designed to use the synergy effect of the polymer matrix and the introduced nanomaterial, which allows meeting the requirements for specific applications in removing contaminants from water [29].

The ion exchange membranes used in electrodialysis processes are very similar to conventional ion exchange resins also in terms of their chemical structure. They are characterized by high selectivity and low resistivity. Such membranes are most often obtained by introducing anionic or cationic groups, respectively, into a pre-formed solid foil, such as a membrane based on styrene-DVB or polysulfone, and then dissolving the modified polymer and, from the solution thus obtained, casting a film forming the membrane [30].

The selection of the appropriate membrane and water purification techniques allows obtaining the desired results. The use of membranes in the treatment of water sources containing anionic micropollutants is most commonly used with RO using filter membranes or ED, especially when separation into monovalent and polyvalent anions is desired. In the case of using NF membranes, it is a consequence of both the size of the ions and the charge exclusion effects, while in ED it is due to the use of ion exchange membranes permeable to monovalent anions [31]. The combination of the advantages of membrane separation with biological reactions in the treatment of surface waters has led to the development of three main types of membrane bioreactors: pressure-differential membrane bioreactors, biological membrane contactors, and pressure-driven membrane bioreactors, biological membrane contactors, and ion exchange membrane bioreactors [32].

Another example of a novel water treatment system is the use of biopolymer-based membranes to remove herbicides from contaminated surface waters. The use of biopolymer membranes with natural polymers (chitosan and alginate) for the removal of widely used herbicides, such as Diquat (DQ), Difenzoquat (DF), and Clomazone (CLO), was investigated [33]. It was found that the alginate-based membranes showed good DQ and DF absorption, mainly due to the possibility of Coulombic interactions between the alginate carboxyl groups and the positive charges of these herbicides. The key determinants of the membrane adsorption capacity are the dissociation constants and the herbicide partition coefficients since higher dissociation constants and lower partition coefficients resulted in higher adsorption. Chitosan/alginate hybrid membranes with a layered structure did not show the best result. However, this type of membrane may be of interest in the context of the adsorption of different herbicides in the various layers of the membrane, e.g., a positively charged herbicide may be adsorbed onto an alginate layer and a negatively charged herbicide may simultaneously be adsorbed into the chitosan layer while being a very effective adsorbent for DQ. Moreover, the pH value of contaminated water may be an important parameter determining the adsorption behavior of the tested herbicides.

Another source of surface water and wastewater contamination can be industrial processes related to metal coating, which is one of the most widely used surface finishing techniques for various parts of the devices. In this process, the surface is coated with the deposition of certain metals, and the process itself is one of the most dangerous industries due to the production of a large amounts of waste chemicals. The wastewater streams generated during the processes taking place in this industry are highly polluted and contain solvents, oils and greases, organic compounds, and heavy metal ionic compounds, such as chromium (Cr), copper (Cu), zinc (Zn), lead (Pb), cadmium (Cd), nickel (Ni), and iron (Fe), in addition to other different cations and anions [34].

Significant pollutants of surface waters, consider ed in the context of their negative impact on aquatic organisms, due to limited dissolution in water, are substances such as oils, including crude oil. In the event of infrastructure failure, tank leakage, and uncontrolled release of oils into waters, it is essential to take action in a place of contamination. One of the actions taken is the use of a barrier, including membrane barriers (Table 1), which allow the separation of the oily substance and the purification of the water.

Various polymer membranes are most commonly used to remove such contaminants, and their operational and practical design and manufacturing aspects are important, ensuring high separation efficiency and purification efficiency. Particular attention should be paid to the careful removal of nickel, chromium, and zinc, due to the harmfulness of these metals and their ions. In the process of removing this type of contamination, it is important to select not only high-performance polymer membranes, but also to select an appropriate treatment process. The most commonly used are reverse osmosis (RO), nanofiltration (NF), ultrafiltration (UF), complexation–ultrafiltration (CUF), microfiltration (MF), polymer inclusion membranes (PIMs), electro-membranes (EMs), hybrid processes, liquid membranes, emulsion liquid membranes (ELMs), and membrane-based solvent extraction [44]. Based on the tests performed, it was found that the polymeric material of the membrane, in order to obtain high separation efficiency, should have certain general characteristics, such as high chemical stability, good formability, reasonable purchase cost, and desirable thermal and mechanical stability. The properties of the isoelectric point and surface charge of membrane materials play an important role in the efficiency of reverse osmosis and nanofiltration processes, while in the case of electro-membranes and liquid membranes, electrical resistance and membrane stability are important [45].

### 2.2. Contamination of the Soil Environment

Soil contamination poses a serious threat to human health. Dangerous and ubiquitous pollution is caused by, among others, heavy metals polluting water, soil, feed, and food [46,47]. Moreover, large amounts of waste and the intensive use of chemicals, especially in agriculture, in recent decades have resulted in a significant threat to ecosystems [48]. The main factor influencing the quality of soil is human activity, incl. operation of mines, storage of industrial or municipal waste. For example, in 2018, according to Eurostat data, 5.2 t of waste were generated per capita [49]. Unfortunately, not all waste is recycled and a significant part of it is deposited in landfills, and in the case of hazardous waste, also in burial grounds. Therefore, soil contamination with CBRN substances mainly occurs near landfills, in areas of intense industrial activity, or in the case of an accident. It should be emphasized that soil contamination has a direct negative impact on human health [50].

Due to the high and constantly increasing level of environmental contamination, there is a need to search for effective techniques and engineering methods for purifying or utilizing sewage and other waste streams that pollute the soil. Depending on the type of contamination, various methods of soil reclamation and protection are used, which can generally be divided into chemical, physical, and biological methods [51].

Depending on the type of pollution and the purpose of the reclamation works, membrane technologies are also used, including geomembranes as barriers to seal landfill areas. Due to the structure and raw materials of which geomembranes are made, these are divided into:

1. Flat membranes made of oxidized asphalts or modified with polymers (poly (vinyl chloride) polyvinyl chloride PVC), or terpolymer obtained from ethylene-propylene-diene rubber monomers (ethylene propylene diene rubber EPDM or high density polyethylene HDPE).
2. Extruded HDPE membranes.

Due to their chemical structure, synthetic geomembranes are resistant to most chemicals and also have good resistance to biological degradation and good mechanical strength. Geomembranes are used in places where protection of the natural environment against contamination is required:

- petrol stations and their storage facilities,
- sewage treatment plants,
- oil boiler rooms,
- rainwater sedimentation tanks,
- hazardous substances reloading yards,
- recycling yards for scrapped vehicles,
- drainage ditches at road bodies,
- sealing municipal waste landfills, and
- broadly understood agrotechnics.

It is very important to choose the right geomembrane material taking into account its application. Multi-criteria decision making (MCDM) provides useful selection tools and has been applied successfully to select geomembranes [52]. Various materials used for the production of geomembranes were tested and it was found that HDPE is the most appropriate. In contrast, PVC is the least useful material for the production of geomembranes. Geomembranes are commonly used to protect soil against leakage of hydrocarbons and other petroleum substances that have a significant impact on the environment and can pose a serious threat to both humans and other forms of life in the polluted environment [53].

Contamination with petroleum hydrocarbons constituting persistent organic pollutants, which include many organic compounds such as polycyclic aromatic hydrocarbons (PAHs), is characterized by high stability, which problematizes their degradation and causes them to remain unchanged in the environment for a long time [54]. The presence of these substances in the ecosystem is usually related to anthropogenic sources that cause significant environmental problems, namely ecological and social disasters around the world. Geomembranes are used to limit leakages from tanks or landfills and to remove and prevent the further spreading of pollutants released during accidents and disasters. Currently, HPDE membranes are most commonly used [7]. This is due to their very good mechanical properties, resistance to chemical and biological degradation, and suitability for operation in various conditions, e.g., in areas with high or low temperatures.

The influence of temperature and soil properties on the penetration process of petroleum hydrocarbons through three different types of geomembranes (HDPE, low density polyethylene LDPE, PVC) was investigated for both laboratory samples and samples after three years of use in the area of low temperatures. The diffusion parameters of the geomembranes were measured at 7 and 14 °C, and the data were combined with previously published test results at 23 °C. PVC geomembranes were also tested at 2 °C. It was found that all the measured parameters decreased with the temperature. Moreover, exposure of LDPE and PVC to cyclic freezing and thawing did not affect the values of diffusion parameters. The diffusion parameters of the HDPE geomembrane collected from the research site at the Resolution Island landfill (an island in the Labrador Sea, located on the south-eastern shore of Baffin Island), after three years of use, were compared with the unaged and unexposed HDPE geomembrane from the same manufacturer, confirm their comparability. On this basis, it was found that the cold climate and cyclic freezing and thawing in the field did not negatively impact the diffusion parameters of the geomembrane. Thus, the research results fully confirmed the usefulness of such protection as a diffusion barrier in the landfill [55].

Geomembranes can be also subjected to elevated temperatures both due to their use in various areas and due to exothermic degradation processes taking place in landfills. Elevated temperatures may reduce the life or effectiveness of geomembranes by accelerating the loss of antioxidants in the geomembranes and degradation of the polymer [56,57]. A case history is provided to illustrate the potential effects of elevated temperatures and time-temperature history on the HDPE geomembrane and associated reduction in service life or performance. Based on laboratory studies and a case study of an aluminum waste landfill, it was found that the usefulness of the geomembrane may be reduced to decades when temperatures reach 60–80 °C, due to degradation processes related to the loss of antioxidants.

The destruction and degradation of membranes may cause unsealing and release of pollutants into the environment [58]. Therefore, the type and nature of membranes should be properly selected depending on the environmental conditions and the purpose of the reclamation (Table 2).

Geomembranes may also be subjected to mechanical damage causing leaks from landfills or other protected places. Minimizing leakage through the geomembrane is very important for environmental protection and water management, and is essential in many industrial applications [66]. Each geomembrane laid, and especially the large-size one, should be tested to avoid any undetected damage. Geomembrane lined containment rooms are designed to contain fluids. It is often difficult to detect leaks that arise, and on occasions, it may take years or decades to detect groundwater contamination. An effective method of leak detection is the electrical leak location (ELL) technology. All ELL methods follow the basic principle of introducing an electric potential through the geomembrane by applying a current source to a power source above the geomembrane and returning the current below it. If there is a break in the geomembrane, then an electric current will flow from the source to the return through the break. It should be added that ELL methods can be divided into two different categories, i.e., exposed geomembrane methods and covered geomembrane methods [67].

The biggest advantage of the covered geomembrane methods is that the measurements are made after the covering material has been laid. This makes it possible to detect the largest leaks. The disadvantage of these methods is that detection sensitivity is extremely dependent on site conditions, liner cross-section, and materials in place, in addition to operator skill and methodology. Based on the evaluation of many commercial geomembranes with the strain hardening modulus, a relationship was found between these moduli and the notched constant tensile load NCTL [68]. This compound provides the basis for the evaluation of the mechanical properties of geomembranes using ASTM D5397 [69].

### 2.3. Groundwater Contamination

Contaminants released into surface water or soil may continue to migrate and determine the quality of groundwater, which is often a source of drinking water. It should be noted that the amount and type of pollutants are influenced not only by the geological basis, type of land, vegetation, and human activity, but also by the hydrological system, water movement, or its intensity in the hydrogeological profile or hydrogeological unit. The movement of groundwater is often diversified, depending on the hydrogeological space and physical conditions, including speed, direction, hydraulic head, and pressure. All individual geochemical processes lead to the diversification of the chemical composition of groundwater. During circulation, mixing of groundwater of different composition occurs. This is a very important process that commonly takes place along the water flow paths, typical for the saturation zone, i.e., the rock layer in which the free spaces (crevices, pores) are completely filled with water. In the feed zone, along the streamline, groundwater mixes with successive portions of water percolating through the aeration zone [70,71]. The chemical composition of groundwater in the drainage zone is usually the result of the composition of waters with different transit times, flowing from different distances, e.g., within a local or regional circuit [70,71,72].

The most important physicochemical reactions taking place in the groundwater environment include: dissolution–precipitation, oxidation–reduction, sorption, and ion exchange. Depending on the conditions under which they occur, these reactions may be reversible or irreversible. In the case of irreversible reactions, the composition of the water is usually determined not by the equilibrium state, but by the kinetics of the reaction. Many processes and reactions take place with varying intensity in different environments. Some of them are, under certain conditions, more important, e.g., for waters in the aeration zone and shallow waters of the saturation zone, they are chemical weathering and dissolution, and when describing the saturation zone, sorption and redox processes [73,74].

In groundwater, electrokinetic and osmotic processes also take place, which consists of the selective migration of aqueous solutions through clay layers that behave as semi-permeable membranes. Such processes are commonly referred to as ultrafiltration or membrane processes. They are also referred to as osmotic processes, sieve effects, or ion filtration. Under certain conditions, they play an important role in shaping the composition of underground waters and deep waters [75].

For the migration of contaminants present in the water environment of the saturation zone, sorption processes occur relatively quickly (e.g., compared to the dissolution processes of most minerals) and the equilibrium stabilizes slowly during redox processes, which run much slower. In the deeper environments of the saturation zone, in conditions of difficult groundwater exchange, the influence of both groups of processes is usually much more visible than in the waters of the aeration zone [76].

The presence of gases such as O_2_, H_2_S, CH_4_, NH_3_, and organic substances in a water-rock medium is of the utmost importance in most redox processes. However, the direction of oxidation–reduction processes in groundwater depends primarily on the content of oxygen, carbon, and organic substances as well as the forms (speciation) of sulfur, nitrogen, iron, and manganese. Most redox reactions take place with the active participation of microorganisms. The strongest reducing properties are exhibited by alkaline and alkaline earth elements. The role of oxidants can also be played by, inter alia, Fe(+3), Mn(+4), S(+6), and N(+5) [76,77]. Among the factors influencing redox reactions, the following are important:

- oxygen content in the water feeding a given aquifer,
- distribution and reactivity of organic matter and other potential reducers in the groundwater reservoir,
- distribution of potential redox buffers in the groundwater reservoir, and
- intensity of groundwater exchange [77].

However, it should be remembered that the presence of contaminants in groundwater in the form of organic, inorganic, radioactive, or microbiological compounds is in most cases a consequence of human activity on the soil surface, the migration of pollutants in the process of infiltration, diffusion, together with rainwater, into groundwater. On the other hand, higher and undesirable concentrations of contamination may be the result of environmental conditions. High concentrations of heavy metals such as Fe and Mn are often the result of a geological basis. In such a situation, actions are taken outside the system to subject water purification processes for drinking or other purposes, e.g., for the brewing industry. Therefore, they do not require taking corrective actions at the place of contamination, but only adaptation to the recipient’s requirements. Thus, membrane systems are subject to different requirements depending on the purpose of remediation. Many different factors must be taken into account when selecting a membrane in the event of contamination. A given solution often has to be dedicated to both the type of pollution, the characteristics of the system to be cleaned, environmental conditions, including the presence of microorganisms, and the purpose of the activities carried out.

Remediation typically involves extracting the groundwater from the well, treating it at various stages of the separation and purification process, and putting the water back underground. Processing can include various steps, including but not limited to, solids filtration, ozonation, air stripping, adsorption, or pH control. Strict water quality standards must be met before water is reintroduced. The rehabilitation process sequence is site-specific and the design of the process must depend on the condition of the aquifers and local regulations. An example is a reclamation carried out by 3M factory [78], the task of which was not only to remove contamination from the water, but also to bring the concentration of dissolved oxygen (DO) in the purified water to the DO concentration of the water in the receiving aquifer, i.e., to a concentration <1–2 mg/L. Membrane degassing (MDG) was used, using 3M™ Liqui-Cel™ 4 × 28 Membrane Contact (MC) membrane contactors and an ultrafiltration system consisting of 3M™ Liqui-Flux™ UF modules as pre-treatment for MCR, with the goal of ultrafiltration membranes were to remove solid particles and colloidal substances, as well as reduce organic carbon and biological pollutants [78].

Metals present in the environment, such as As, Cd, Cu, and Hg, are very dangerous to human health and environmental safety. However, their removal from groundwater is very difficult due to the presence of metals in various oxidation states and the formation of various compounds, depending on the physicochemical properties of the environment. Unfortunately, the development of industry, including plant protection products, has resulted in more and more of these pollutants in groundwater. Many membrane techniques are used to remove them, including nanofiltration, reverse osmosis, the effectiveness of which depends on, among others, the pH of the solution (Table 3).

One of the increasingly used techniques for removing metals, including arsenic, is nanofiltration. However, it should be noted that performance, water permeability, and resistance to contamination may vary depending on the type of membrane. In the case of NF/RO membranes used to remove arsenic and depending on the pH of the environment, it may be from 5% to 99% [92,93]. Tanne et al. [94] investigated the effect of membrane pore size and surface properties on membrane performance. The study included four fully aromatic polyamide membranes with different physicochemical properties, including three commercially available NF membranes, i.e., NF90, ESNA1, and ESNA1-LF2-LD and one commercially unavailable membrane M#1. All four membranes were hydrophilic, with the M#1 membrane being the most hydrophilic, with the support layer of a polysulfone or polyamide active layer different from the other membranes and with the smallest pores. The research results showed that the pore size and surface charge, hydrophobicity, thickness of the active layer and roughness are important factors for the membrane operation. These properties can significantly affect water permeability and/or arsenate rejection. On the other hand, the reaction environment was also important, including the pH value of the solution. The most optimal conditions for the rejection of arsenate were obtained at about neutral pH [94].

Modifications with the use of nanoparticles with high oxidation potential, such as zero-valence iron nanoparticles (nZVI), are widely used. The use of nZVI integrated into the structures of membranes allows to prevent agglomeration and reduces the likelihood of secondary pollution. On the other hand, however, their mechanical strength is limited, which is critical for the long-term operation and regeneration of membranes [95]. Ren et al. [95] developed a high molecular weight double crosslinking method to improve the mechanical strength of polymeric electro-spun nanofiber membranes. Polyacrylic acid is one of the main high molecular weight (PAA, Mw = 450,000) polymers of nZVI [95,96,97] immobilization that has been double cross-linked by adding polyvinyl alcohol (PVA) and Fe(II) or Fe(III). PVA was added as a covalent cross-linking agent, while the iron particles acted as an ionic linker. The obtained results indicated that the Fe(III) -based PVA-PAA-nZVI membrane showed a high potential for the long-term filtration process and allowed for a higher degree of Cd ion removal from groundwater [95].

Groundwater more and more often, apart from metals, contains higher and higher concentrations of nutrients that come from crops, landfilled waste, or leachate. Nutrients migrate with rainwater into the soil, undergoing transformation, depending on the chemical, physical and biological characteristics of the environment. Contamination of groundwater with nutrients poses a threat to human health, as in many areas these waters constitute a reservoir of drinking water. Therefore, a lot of work is focused on the removal of various forms of nitrogen and phosphorus. Among others, Zou et al. [98] carried out studies on NO_3_^-^ removal using a PA nanofiltration membrane modified with poly(sodium 4-styrene sulfonate) (PSS). The research results show that the most optimal system, for which the nitrate rejection rate of 88.8% was obtained, was at the PSS concentration of 1.5 mg/L and the permeate flux of 27.0 L/m^2^·h. The influence of the initial nitrate concentration and solution pH on the effectiveness of nitrate removal through the modified NF membrane was investigated. It was found that the nitrate rejection rate was further enhanced by PA/PSS at a lower pH, while the membrane permeate flux improved as the pH increased. The initial concentration of nitrates had little effect on both the rate of nitrate rejection and the discharge from the membrane [98].

In addition to nutrients, pesticides also pollute groundwater. Plattner et al. [99] carried out work to remove five pesticides, i.e., Phorate (O,O-diethylS-ethylthiomethyl phosphorodithioate), Parathion-methyl (O,O-dimethylO-4-nitrophenyl phosphorothioate), Atrazine (6-chloro-N2-ethyl-N4-isopropyl-1,3,5-triazine-2,4-diamine), Dichlorvos (2,2-dichlorovinyl dimethyl phosphate), and Clofibric acid (2-(4-chlorophenoxy)-2-methylpropanoic acid), in the brackish model groundwater solution using a laboratory direct membrane distillation (DCMD) system. It was found that the effectiveness of pesticide treatment with DCMD depends mainly on the properties of these compounds. Pesticides with low hydrophobic properties and low vapor pressure showed a high rejection rate (70–99%), while compounds with high vapor pressure or high hydrophobicity reduced rejection (30–50%) with a water recovery of 75%. It was also found that organic (humic acid) and inorganic ions (Na^+^, Ca^2+^, Mg^2+^, Cl^−^ and SO_4_^2−^) do not significantly affect the degree of pesticide rejection by DCMD [99].

Šír et al. [100] also started work on the removal of chlorinated pesticides, mainly α-HCH, β-HCH, γ-HCH, HCB, DDE, DDD, and DDT from groundwater. A LAB M-20 membrane, a small-scale separation test unit, with a LabStack M20 membrane module, with a nominal capacity of 30 dm^3^/h, at a maximum pressure of 6.0 MPa, was used for the tests. Due to the requirement of high-quality permeate, RO98pHt reverse osmosis membranes (thin-layer polypropylene composite) were used. Observed removal rates of chlorinated pesticides ranged from 98.4% to 99.7% in the presence of high salt content. The separation efficiency was slightly higher for DDT and its derivatives than for HCH isomers [100].

Ainscough et al. [101] started work on removing chlorinated volatile and non-volatile organic compounds from groundwater, such as trichlorethylene (TCE), tetrachlorethylene (PCE), cis-1,2-dichloroethylene (DCE), 2,2-dichloropropane (DCP), and vinyl chloride (VC). Three types of membranes were used: ceramic microfiltration membrane Pall Membralox (made of α-Al_2_O_3_), four polymer nanofiltration membranes (namely DK and DL membranes from GE Osmonics, NF90 and NF270 from Dow Filmtec), five reverse osmosis membranes (AK and AG-GE Osmonics, BW30, BW30LE, and BW30XFR from Dow Filmtec, fouling resistant). The use of nanofiltration membranes allowed for the reduction of pollutants at the level of almost 100% (with the exception of VOCs), with a maximum operating pressure of up to 3 bar. On the other hand, microfiltration membranes and reverse osmosis membranes did not give positive results, which was probably due to the adsorption of hydrophobic VOC compounds on the membranes and the inability to pass the solution through the membrane matrix [101].

The problem of groundwater contamination with chlorinated organic compounds such as trichlorethylene (TCE), tetrachlorethylene (PCE), polychlorinated biphenyl (PCB) and carbon tetrachloride (CTC) was also addressed by Wan [102,103]. In order to remove the contamination, he used commercial microfiltration membranes made of polyvinylidene fluoride (PVDF), which were functionalized with poly (acrylic acid) (PAA) or poly(methacrylic acid) (PMAA). Functionalization caused deprotonation of hydroxyl groups, as a result of which the membranes became hydrophilic at pH > pKa. In addition, Pd-Fe nanoparticles with sizes <20 nm were embedded in the pores of the membranes. The membranes thus obtained were used in the removal of organic contaminants from the Louisville, KY landfill. The obtained results indicate that the modified Pd/Fe-PMAA-PVDF membranes under laboratory conditions allowed for the degradation of 3,3′,4,4′,5-pentachlorobiphenyl (PCB-126) as a reference substance, even up to 96% in a shorter period of time than 15 s. In the case of other compounds, the reaction rate or the efficiency of removal of impurities varied, and in the case of, for example, TCE and CTC compounds, a reduction of about 90% and 85%, respectively, was obtained in 2.2 s. The verification of the membranes, carried out in the field conditions in groundwater, allowed for a significant reduction of pollutants. The purified waters remained 0.1% CTC, 12% TCE, and 18% TCE with a residence time of 2.4 s. Based on the obtained results, it was found that the degradation rate changed in the series: carbon tetrachloride > trichlorethylene > tetrachlorethylene > chloroform [102,103].

## 3. Conclusions

Research on the use of polymer membranes to counteract the risk of environmental contamination is undergoing constant development due to the possibility of using this technology to purify a whole range of surface and ground waters as well as the soil environment, which is particularly important from the point of view of environmental protection. In addition, the ability to recover valuable natural resources draws attention to important economic aspects. Increased interest in the use of this type of technology is also the result of the growing environmental awareness of societies. Polymer membrane processes do not require dosing of chemicals and do not transform pollutants, saving resources, energy, and human labor. In view of the above facts, there is a continuous and intensive development of research on obtaining more effective methods, allowing the modification of polymer membranes, and at the same time changing their physicochemical properties.

There are many advantages of using polymer membranes in environmental protection. First of all, compared to many other traditional techniques and processes, e.g., distillation, polymer membranes use less energy, as well as fewer raw materials and operating personnel. It should also be noted that the use of membrane techniques by industry may contribute to increasing resources (mainly water) and the reduction of the amount of solid and liquid waste generated in the production process, and thus allows for obtaining tangible economic benefits. Water purified by membrane processes can return to production and does not remain in the soil and groundwater, poisoning these resources.

In conclusion, the use of polymer membranes seems to be justified due to many factors, chiefly technical and economic (at least relatively low operating costs), as well as the properties of their easy adaptation in the environment. The introduction of membrane techniques into widespread use is considered to be the right step in the field of environmental protection. It is also assessed that these technologies in water and soil treatment applications are currently among the best available technologies and that they make a beneficial contribution to environmental sustainability. Some of them require relatively high investment outlays. The applicable legal regulations and economic instruments of environmental policy should support the use of these techniques in industry and the economy. For example, the penalties related to the direct introduction of pollutants into the environment are high and it remains necessary to invest in environmental protection, including investments in polymer membrane techniques.

## Figures and Tables

**Table 1:** Examples of the use of membranes to remove oil from surface water.

Technique	Type of Membrane	Conditions	Ref.
UF	PPSU/TBF	Transmembrane pressure of 1 bar; a flow rate of 300 mL/min along the lumen side; a velocity range of 2.58–2.81 m/s	[35]
Gravity-driven filtration	NiCo-LDH/PVDF composite	Glass sand core filter device; water-in-oil emulsions (soybean oil, petroleum ether, 1,2-dichloroethane, n-hexadecane)—the volume ratio of 1:99	[36]
Filtration	APTES@PVDF/GO	Polymerization with ATRP; a volume ratio of organics and water: 1:99; the pressure of 0.05 MPa; complex environments, such as 2 M HCl, 2 M NaOH and saturated NaCl; permeation flux 1000 ± 44 L/m^2^·h·bar	[37]
Gravity-driven filtration	nanofibrous PVDF membrane	Permeability 88 166 ± 652 L/m^2^·h·bar; water-in-oil emulsions (chloroform, toluene, dichloromethane and high viscosity oils: D4 and D5)	[38]
Photoreactor	TiO_2_-NPs/PVDF-TrFE	The flow rate 100.8 L/h; pH = 4–5.5; oily industrial wastewater	[39]
Separation	SiO_2_-NPs/PVDF	The pressure of 0.9 bar; fluxes of over 10,000 L/m^2^ h	[40]
RO	PES or PVDF (EM006, ES209, ES625, FP100, FP200)	The cross flow velocity 2 m/s; operating pressure 60 bar; crossflow membrane sequencing batch reactor inoculated with isolated tropical halophilic microorganisms	[41]
VDF system	CS–SiO_2_–GA composite/PVDF	Separation area ~1.6 cm^2^; the pressure 0.03 MPa.	[42]
Separation	TiO_2_-NP/PVDF	Pressure difference of 0.09 MPa; separation area 1.77 cm^2^; the permeation flux for SDS/oil/H_2_O emulsion (oil: petroleum ether; n-hexadecane; 1,3,5-trimethylbenzene; diesel oil): 428 L/m^2^∙h, 605 L/m^2^∙h, 524 L/m^2^∙h, 382 L/m^2^∙h respectively	[43]

PPSU—sulfonated polyphenylenesulfone polymer; TBF—triangle-shape tri-bore hollow fiber membranes; NiCo-LDH—nickel cobalt layered double hydroxide; PVDF—the polydopamine modified polyvinylidenefluoride membrane; APTES—3-aminopropyltriethoxysilane; ATPR—atomic transfer radical polymerization; PVDF—poly(vinylidenefluoride); TrFE—trifluoro ethylene; PSH—poly(3-(N-2-methacryloxyethyl-N,N-dimethyl)ammonatopropanesultone)-co-2-hydroxyethyl methacrylate; CS—chitosan; GA—glutaraldehyde; VDF—a vacuum driven filtration system; SDS—Sodium Dodecyl Sulfate.

**Table 2:** Examples of the use of membrane techniques to soil reclamation.

Type of Membrane	Pollution	Conditions	Ref.
HDPE	BTEX: benzene, toluene, ethylbenzene, xylenes	a landfill site in the Canadian Arctic; temperature: 2, 7, 14 °C; geomembranes below the 2 m thick soil; lowering the temperature of the geomembrane reduces the amount of pollution transport increase	[55]
HDPE	The municipal landfill leachate	2.0 mm of nominal thickness of geomembrane; the nature of the leachate determines the strength and efficiency of the membrane	[59]
HDPE	fluid retention of leaching in sanitary landfills	influence of different purge gases at different heating rates (5, 10, 15 and 20 °C/min); deformation of geomembranes under the influence of temperature, environmental chemistry, pressure and heat prevailing on geomembranes, deposition of residues in geomembranes	[60]
HDPE	Landfill	1.5 mm thick; vertical pressure of 250 kPa; temperature 85 °C; coarse gravel determines cracks and dents (stress crack)	[61]
HDPE	Municipal solid waste leachates	temperature: 22, 40, 55, 70, 85 and 95°C; salts and VFA have a significant influence on the mechanical properties of the geomembrane (especially resistance to stress cracking)	[62]
LLDPE/GCL	Insulation tailings	peat bog—up to 5.5 m thick; glacial till—thickness from 0.5 to 3.1 m beneath the perimeter dam wall; bedrock—comprising Waulsortian limestone (30–80 m thick)	[63]
PVDF/TiO_2_	Boron removal from landfill leachates	achieving a homogeneous TiO_2_ surface under defined loading is critical to achieving good boron rejection results	[64]
BPM/ED	Cr(III)/Cr(VI)	Effectiveness depends on: cell voltage, soil pH, current efficiency, and specific energy consumption; the optimal current density 2.0 mA/cm^2^;	[65]

HDPE—highdensity polyethylene; VFA—volatile fatty acid; LLDPE—linear low density polyethylene; GCL—geosynthetic clay liner; BPM—bipolar membrane—the alkaline stable poly(terphenyl) anion exchange membrane; ED—electrodialysis.

**Table 3:** Examples of the use of membrane techniques to remove metals from groundwater.

Technique	Type of Membrane	Metal	Conditions	Ref.
RO	ES-10, NTR-729HF	As, Sb	pH = 3–10, the removals of As(V) and Sb(V) are much higher than those of As(III) and Sb(III)	[79]
NF/RO	ES-10 and HS5110/HR3155	As	NF: pressure 0.2 to 0.7 MPa/RO: pressure 4 MPa	[80]
NF	NF90–4040	Cr, As	pH = 9, temp. 45 °C, pressure 3.1 MPa	[81]
NF	UiO-66 (Zr-MOF)/TFN	Se, As	1,15 LMH/MPa	[82]
NF	The P[MPC-co-AEMA] co-polymer/	Se, As	0,85 LMH/MPa	[83]
VF	PVDF with melanin nanoparticles from the marine bacterium *Pseudomonas stutzeri*	Hg, Cu, Cr, Pb	45 °C; pH = 3 for Cr and pH = 5 for other metals; flow rate of 0.5 mL/min	[84]
MEF	M-I	Cu, Pb, Cd	10-layer filtration; pH = 6.5–8.5; flow rates of feed 30 L/h	[85]
MF	PTFE/HPAMAM	Cu	operating pressure 25 kPa; the flux 63,579 L/m^2^ h	[86]
EUF	PAN—Osmonic 100 kDa UF	As	an averaged crossflow velocity of 0.1 m/s; pressure 98 kPa	[87]
NF, UF	PA (for NF: Koch; for UF: Osmonics)	Fe, Mn	0.5 MPa, pH = 3–11	[88]
NF/RO	Desal AG-2540 RO,TFC-ULP-2540 RO and TFC-SR2-2540 NF	Sr	applied pressure 0.10–0.15 MPa, pH = 3–6	[89]
NF	PEM: PDADMACand PSS on PA	Mg, Sr, Ca, Ba	low ionic strength conditions (e.g., <50 mM NaCl as a background electrolyte); 0.345 MPa; crossflow velocity 21.4 cm/s; 25 °C.	[90]
Hybrid: Oxidation/MF	tubular Kerasep^®^ ceramic membranę	Fe	Oxidation: 0.07 MPa; 20–22 °C; MF: tangential velocity 3.2 m/s; transmembrane pressure 0.06–0.3 MPa; pH = 6.8–7.2; 20–22 °C	[91]

MOF—metal-organic framework; TFC—thin-film composite; LMH—L/m^2^·h, P[MPC-co-AEMA] co-polymer-2-methacryloyloxyethyl phosphorylcholine (MPC)-co-2-aminoethyl methacrylate (AEMA); PVDF—polyvinylidene fluoride; VF—vacuum filtration; MEF—micellar enhanced filtration; M-I—nanofiber membrane prepared from chloridized polyvinyl chloride by high-voltage electrospinning process; HPAMAM—hyperbranched poly(amidoamine) (the hydrophilic chelating agent); EUF—Electro-ultrafiltration; PA—polyamide; PEM—polyelectrolyte multilayer membrane; PDADMAC—poly(diallyldimethylammonium chloride); PSS—(poly(sodium 4-styrenesulfonate).

## Data Availability

Not applicable.
